# Allergic Contact Dermatitis Is Associated with Significant Oxidative Stress

**DOI:** 10.1155/2014/415638

**Published:** 2014-08-11

**Authors:** S. Kaur, K. Zilmer, V. Leping, M. Zilmer

**Affiliations:** ^1^Clinic of Dermatology, University of Tartu, 31 Raja Street, 50417 Tartu, Estonia; ^2^Institute of Biomedicine and Translational Medicine, Department of Biochemistry, the Centre of Excellence for Translational Medicine, University of Tartu, 19 Ravila Street, 50411 Tartu, Estonia; ^3^Institute of Computer Science, University of Tartu, 2 J. Liivi Street, 50409 Tartu, Estonia

## Abstract

*Background*. Research has confirmed the involvement of oxidative stress (OxS) in allergic contact dermatitis whilst other inflammation-related biomarkers have been less studied. *Objective*. To evaluate systemic levels of selected inflammatory markers, OxS indices and adipokines as well as their associations in allergic contact dermatitis. *Methods*. In 40 patients, interleukin- (IL-) 6, monocyte chemoattractant protein (MCP-1), and IL-10 levels were measured in sera with the Evidence Investigator Cytokine & Growth factors High-Sensitivity Array, total peroxide concentration (TPX) and total antioxidant capacity (TAC) by means of spectrophotometry, and the plasma concentrations of adiponectin and leptin by the quantitative sandwich enzyme immunoassay technique. *Results*. TNF-*α* level (*P* < 0.01) and TPX (*P* < 0.0001) were increased whilst IL-10 (*P* < 0.05) and TAC (*P* < 0.0001) were decreased in the patients as compared to controls. Correlation and multiple linear regression analysis identified both, TPX and TAC (inversely), as possible independent markers for evaluating allergic contact dermatitis. Adiponectin level in patients was increased (*P* < 0.0001), but neither adiponectin nor leptin correlated significantly with the biomarkers of inflammation or OxS. *Conclusion*. OxS parameters, especially TPX and OSI, reflect the degree of systemic inflammation associated with allergic contact dermatitis in the best way. The relation between OxS and adiponectin level warrants further studies.

## 1. Introduction

Inflammation and oxidative stress (OxS), the latter defined as an overproduction of reactive oxygen as well as nitrogen species (ROS and RSN, resp.) with concomitant deficiency of antioxidative defenses of the body [1, 2], have been found to be inextricably connected in physiological as well as disease states [1]. In allergic contact dermatitis, elevated systemic levels of interleukin- (IL-) 6, IL-1, and tumor necrosis factor (TNF)-*α* have been found [3, 4], and ROS are proposed to participate in the initial allergen sensitization as well as in the development of pathogenic allergic responses [5].

Both, OxS and inflammatory mediators such as cytokines and C-reactive protein (CRP) have an influence on the adipokine status [6–8]. Adiponectin, a cytokine produced solely by adipose tissue, possesses antidiabetic, antiatherogenic, and potent anti-inflammatory activities [9, 10]. In accordance with its anti-inflammatory character, adiponectin levels are increased, rather than decreased, in a number of chronic inflammatory and autoimmune diseases [11]. Inverse correlations of adiponectin with markers of OxS and inflammation have been previously found [7, 9, 12]. The second most important adipokine leptin is known primarily by its ability to regulate food intake and energy expenditure [13]. Leptin has predominantly proinflammatory functions [11], promoting, for example, the activation and production of oxidative burst of inflammatory cells [13].

The current study was undertaken to concurrently examine selective biomarkers of inflammation, OxS, and inflammation-related adipokines as well as their associations in patients with acute/subacute allergic contact dermatitis involving approximately 5% of body surface.

## 2. Methods

Patients for this study were recruited from the Clinic of Dermatology of Tartu University, Estonia. The study involved 40 patients (6 male, 34 female, age 23–67 years, mean age 40.7 ± 13.0 years, mean BMI 25.3 ± 5.5 kg/m^2^) who came to our clinic for patch testing due to acute/subacute allergic contact dermatitis, confined mostly to their hands or face (23 and 11 patients, resp.). Blood from the patients' antecubital vein was collected on the fifth day of routine patch testing after overnight fast. At the time of blood collection, 31 patients had one or more positive patch test results on their back, including 19 patients with positive patch test result to 5% nickel sulphate, nine to 0.01% methylisothiazolinone and eight patients to 1% formaldehyde. The remaining nine patients had given positive patch test results in a previous testing, and current dermatitis was associable with their contact allergy. All patients were tested with European standard series purchased from Hal Allergie GMBH, Düsseldorf, Germany, and allergens were applied on the skin with Finn Chambers on Scanpor (SmartPractice, Phoenix, USA). The patients were not treated with systemic corticosteroids at least for a month and any local corticosteroids were avoided for 24 hours before patch testing. The patients did not have active infections and concomitant chronic diseases as determined by the anamnesis, clinical assessment, and laboratory measurement of blood count, blood glucose level (>5.5 mmol/L), and total cholesterol (>6.9 mmol/L) at the time of recruitment. Collected blood samples were centrifuged at 1500 ×g for 10 min and the serum was divided into aliquots and stored at −70°C for subsequent analysis.

The results obtained from the patients were compared against the data of age-matched normal weight healthy subjects from the database of the Institute of Biochemistry of Tartu University. Cytokine levels were compared with a control group (Co) consisting of 40 healthy subjects (14 male, 26 female, age 38.3 ± 11.8 years, range 19–58 years, mean BMI 24.8 ± 2.7 kg/m^2^), OxS parameters with the data of 40 healthy individuals (25 male, 15 female, age 39.5 ± 10.6 years, range 20–58 years, mean BMI 23.8 ± 3.4 kg/m^2^), adiponectin level with its levels in 51 healthy subjects (19 male, 32 female, age 37.1 ± 12.00 years, range 21–53 years, mean BMI 23.2 ± 3.9 kg/m^2^), and leptin level with the data of 20 healthy women (age 34.7 ± 11.4 years, range 20–60 years, mean BMI 21.5 ± 1.4 kg/m^2^). The study was approved by the Ethics Committee of the Faculty of Medicine of the University of Tartuand conducted after obtaining a signed consent from each participant.

### 2.1. Measurement of Adiponectin, Leptin, and Cytokine Levels

The plasma concentrations of adiponectin and leptin were analyzed by a quantitative sandwich enzyme immunoassay technique, using commercially available kits (R&D Systems, Minneapolis, MN, USA).

IL-6, monocyte chemoattractant protein (MCP-1) and IL-10 levels were measured in sera with the Evidence Investigator Cytokine & Growth factors High-Sensitivity Array (CTK HS Cat. number EV 3623 RANDOX Laboratories Ltd., Crumlin, United Kingdom) according to the manufacturer's protocol. Assay sensitivity varied from 0.12 pg/L to 2.12 pg/L, depending on the analyte. The reproducibility of the assay for an individual cytokine was determined using the quality controls provided with the kit.

HsCRP was analyzed by immunoturbimetry method at the Laboratory of Biochemistry of the Tartu University Hospital. The level of hsCRP 1.0 mg/L was used as a cut-off point to define a higher inflammatory status.

### 2.2. Measurement of Total Peroxide Concentration

Total peroxide concentration of samples was determined using* OXYSTAT* Assay Kit Cat. number BI-5007 (Biomedica Gruppe, Biomedica Medizinprodukte GmbH & Co Kg, Wien). The kit detects peroxide concentrations based on the reaction of biological peroxides with peroxidase and a subsequent color-reaction using tetramethylbenzidine (TMB) as substrate. The colored liquid is measured photometrically at 450 nm, using ELISA plate reader Photometer Sunrise (Tecan Austria GmbH, Salzburg). The concentration is stated as H_2_O_2_-equivalents (*μ*mol/L).

### 2.3. Assessment of Total Antioxidant Capacity

The basic principle of the method is that a colourless molecule, reduced 2,2-azino-bis-3-ethylbenzothiazoline-6-sulfonic acid (ABTS), is oxidized to a characteristic blue-green ABTS, using hydrogen peroxide in acidic medium (the acetate buffer 30 mml/L pH 3,6) [14]. When the coloured ABTS is mixed with any substance, that can be oxidized, it is reduced to its original colourless ABTS form again. The ABTS is decolorized by antioxidants according to their concentrations and antioxidant capacities. This change in colour is measured as a change in absorbance at 660 nm. The reaction rate was calibrated with Trolox. The results are expressed in mmol Trolox equivalent/L. Within- and between-batch precision data obtained by TAC method were 2.5% and 2.9%, respectively.

### 2.4. Oxidative Stress Index

Percent ratio of the total peroxide concentration of plasma or other biological fluids (TPX) to the total antioxidant capacity of plasma or other biological fluids (TAC) is accepted as oxidative stress index (OSI), an indicator of the degree of oxidative stress [15]. According to this, we calculated OSI as the ratio of TPX (*µ*mol/L) to TAC (*µ*mol Trolox equivalent/L) × 100.

### 2.5. Statistical Analysis

Statistical analysis was performed using SAS 9.2 (SAS Institute Inc. NC, Cary, USA). All data are presented as mean ± standard deviation, and statistical significance was established as *P* < 0.05. A comparison between variables was assessed by the independent sample *t*-test. Correlations between parameters were assessed using bivariate correlation analysis (Pearson correlation coefficient) and multiple regression analysis.

## 3. Results

### 3.1. Inflammatory and OxS Markers in Patients and Healthy Subjects

As shown in Table 1, the patients had significantly increased TNF-*α* serum levels whilst the increase in MCP-1 remained below the threshold of statistical significance (*P* < 0.01 and *P* = 0.059, resp.). IL-10 levels in the patients were decreased (*P* < 0.05; Table 1). The mean concentration of hsCRP in the patients was 1.96 ± 2.60 mg/L, that is, not significantly different from the generally accepted maximum borderline value 1.0 mg/L.

The most noticeable differences between the patients and Co were established in OxS markers levels: TPX in the patients was significantly increased while TAC was decreased (*P* < 0.0001 in both) (Table 1). OSI, the percent ratio of TPX to TAC, was also significantly increased in the patients (*P* < 0.0001).

### 3.2. The Relations between Markers of Inflammation and OxS

To confirm the possibility that inflammatory status is related to the diagnosis of allergic contact dermatitis, Pearson correlation coefficients were calculated, using combined patients' and Co data from Table 1. Significant positive correlations with the diagnosis of allergic contact dermatitis were found for TNF-*α* (*r* = 0.290, *P* < 0.01), MCP-1 (*r* = 0.312, *P* < 0.01), TPX (*r* = 0.769, *P* < 0.0001), and OSI (*r* = 0.769, *P* < 0.0001), and negative correlations for IL-10 (*r* = −0.255, *P* < 0.05), and TAC (*r* = −0.485, *P* < 0.0001). Further multiple linear regression analysis starting with all covariates, for example, age, gender, BMI, TPX, TAC, and OSI also, revealed significant positive correlation of the diagnosis of allergic contact dermatitis with TPX (*r* = 0.591, *P* < 0.0001) and inverse correlation with TAC level (*r* = −0.635, *P* = 0.006).

**Figure 1:**
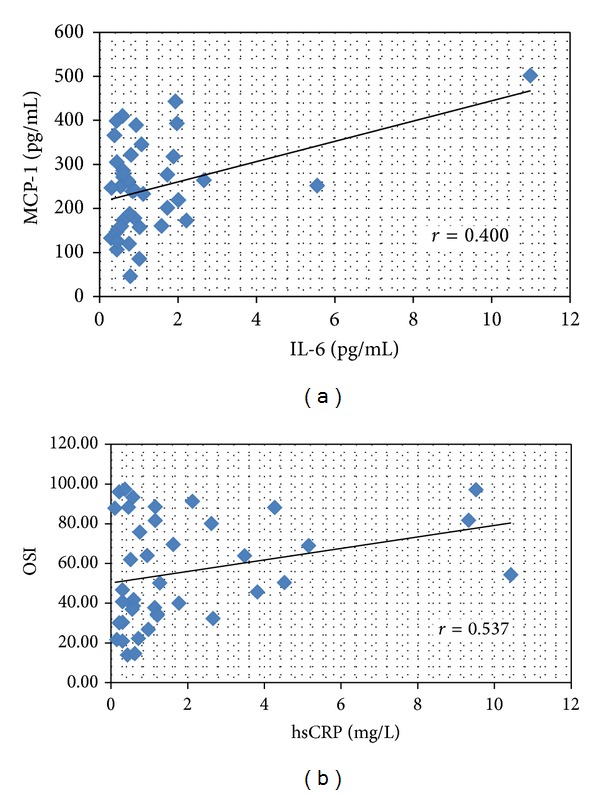
Correlations between inflammatory and oxidative stress markers in patients with acute/subacute allergic contact dermatitis approximately on 5% of body surface. MCP: monocyte chemoattractant protein; IL: interleukin; OSI: oxidative stress index; hsCRP: high sensitive C-reactive protein; TNF: tumor necrosis factor; TAC: total antioxidant capacity; TE: Trolox equivalent; TPX: total peroxide concentration.

We further analyzed the correlations between markers of inflammation and OxS in the group of patients and established relevant positive correlations between MCP-1 and IL-6 levels (*r* = 0.400, Figure 1(a)) as well as between hsCRP and OSI (*r* = 0.537; Figure 1(b)).

**Figure 2:**
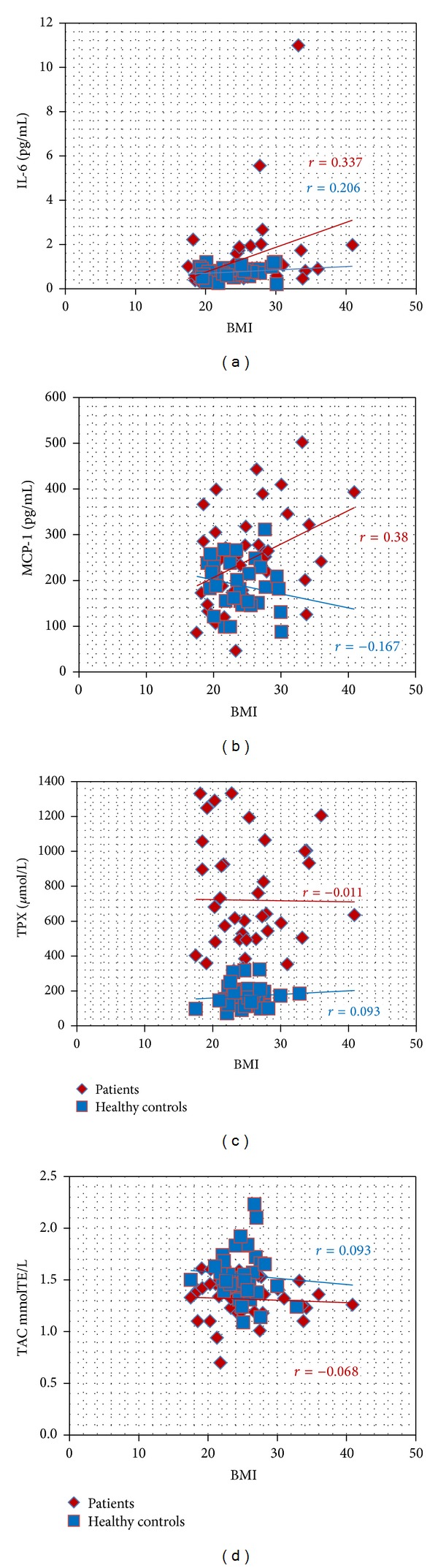
Scatterplot of correlation analysis of IL-6, MCP-1, TPX, and TAC with BMI in patients with allergic contact dermatitis and healthy controls. IL: interleukin; MCP: monocyte chemoattractant protein; TPX: total peroxide concentration; TAC: total antioxidant capacity; TE: Trolox equivalent; BMI: body mass index.

TNF-*α* level, which was most significantly increased in the patients, correlated neither with other inflammatory markers nor the indices of OxS. Both MCP-1 and IL-6 correlated positively with patients' BMI (*r* = 0.380, and *r* = 0.337, resp., Table 2). Therefore, we continued to follow the relationship between inflammatory markers and BMI, including the analysis the patients and a corresponding Co. As shown in Table 2, subjects' BMI was positively correlated with the age (*r* = 0.340) and inflammatory markers IL-6 and MCP-1 (*r* = 0.327, and *r* = 0.295, resp., Figures 2(a) and 2(b)). At the same time, BMI was not correlated to the OxS markers in the patients and Co analyzed together (Table 2, Figures 2(c) and 2(d)). Correlation coefficients between age/TPX and age/TAC were also insignificant (−0.059, and 0.034, resp.).

Other significant relations in patients and Co analyzed as whole were strong positive correlations between IL-6 and MCP-1, as well as inverse correlation between TPX and TAC (Table 2).

### 3.3. Adiponectin and Leptin Levels and Their Associations with the Biomarkers of Inflammation and OxS

Plasma concentration of adiponectin was significantly higher in the patients, compared with the mean value of 51 healthy normal weight controls with no statistically significant differences according to their age and gender (11555 ± 4378 ng/mL and 6081 ± 2422 ng/mL, *P* < 0.0001). The levels of plasma adiponectin in the patients were inversely but nonsignificantly related to the BMI (*r* = −0.225, *P* = 0.16). We found no correlations of adiponectin level with any markers of inflammation or OxS in the patients except an inverse correlation between adiponectin and TPX (*r* = −0.300, *P* = 0.06) that, however, was only close to statistically significant relationship.

As leptin level in women exceeds the values in men two-three times [16] and our sample was predominantly female (85.0%), we calculated the mean leptin level in 34 female patients and compared it to the level in 20 female Co. The obtained values were significantly different (13267 ± 10435 pg/mL, and 7318 ± 3685 pg/mL, resp., *P* < 0.01). On the other hand, blood leptin level positively correlates with body weight [9, 11]. With the adjustment for BMI, which was different in our patients and Co (25.3 ± 5.5 kg/m^2^, and 21.5 ± 1.4 kg/m^2^, *P* = 0.0001), the difference in leptin levels between female patients and Co became insignificant. The correlation of leptin level with BMI had only borderline significance (*r* = 0.239, *P* = 0.066) when analyzed in the patients and Co as whole (*n* = 60). There were no significant correlations between leptin levels and biomarkers of inflammation and OxS.

## 4. Discussion

The most remarkable finding of this study was that acute/subacute skin inflammation consisting of inflammatory lesions on the face or hands and positive patch test reaction site on the patients' back brought about considerable OxS.

Earlier studies have documented the rise in serum levels of several proinflammatory factors after exposure to contact allergens. The increase of TNF-*α* and, to some extent, MCP-1 levels in our patients is in concordance with the results obtained in patients with parthenium dermatitis [17] and trichloroethylene-induced hypersensitivity [18] and might be explained by the activities of activated T cells [19]. In case of inflammation, TNF-*α*, which is present in healthy skin, is additionally synthesized by activated macrophages, T cells, and keratinocytes and released into circulation [19]. Furthermore, contact allergens and irritants can directly induce the expression of both TNF-*α* and MCP-1 [20]. For example, Martín et al. (2003) have demonstrated the expression of MCP-1 by basal keratinocytes and isolated dermal cells at 10 hours after antigen challenge, paralleled by dermal accumulation of mononuclear cells [21]. TNF-*α* stimulates the production of several other cyto- and chemokines, including MCP-1, by fibroblasts, endothelial cells, macrophages, and dendritic cells [21].

Though allergic contact dermatitis has been mainly associated with Th1/Th17 phenotypes, Th2-type regulatory cytokines such as IL-10 may have an important role in the downregulation of contact hypersensitivity reactions [3]. Therefore, decreased IL-10 concentrations, found in this and some previous studies [17, 22], might indicate the utilization of this cytokine in downregulation of allergic contact dermatitis.

Several inflammatory markers, including TNF-*α* and MCP-1, have been correlated with body weight [11, 23]. We found similar correlations for MCP-1 and IL-6; this demonstrates the decrease of their importance as markers of the activity of the inflammation in allergic contact dermatitis. At the same time, OxS parameters did not depend on patients' BMI.

The measurement of TPX and TAC has been currently effectively used to characterize both sides of OxS compendiously, including several conditions not related to infections or inflammation, for example, white-coat hypertension [24] and major depression [25]. In addition, the ratio of TPX to TAC (OSI) gives a single numerical value to evaluate the degree of OxS [15]. Our study of patients with restricted allergic contact dermatitis demonstrated a significant increase in the oxidants pool and decrease in antioxidative capacity; the latter can be explained by the consumption of radical-scavenging antioxidants due to increased free radical amounts [15]. The prevalence of OxS indices over inflammatory is in agreement with the concept that OxS may be the starter point in the pathogenesis of allergic contact dermatitis, leading to the activation of transcription factors and signaling pathways and further synthesis of inflammatory cytokines [4, 5]. Some recent studies have confirmed the ability of contact allergens to induce OxS pathway in keratinocytes [26, 27]. Our own previous study showed the decrease in antioxidants pool of the skin, evidenced by increased oxidized glutathione/reduced glutathione ratio in positive to 5% nickel sulphate patch test site [28]. The presence of contact allergen-related OxS was also confirmed by Gangemi et al. (2009) who found increased serum concentrations of nitrosylated proteins (biomarkers of OxS) in nickel-allergic female patients after oral nickel challenge [29]. Considering all the above-mentioned facts, TPX may be the best and earliest marker of systemic changes occurring in the body after allergen challenge and before the visible inflammation. This marker could be used in the evaluation of treatment results when developing new anti-inflammatory drugs or cosmetic products.

Adiponectin and leptin, the most abundant products of the adipose tissue, have considerable effects on metabolic, inflammatory, and immune responses [16]. Well-recognized activities of adiponectin include the induction of anti-inflammatory mediators such as IL-10 and IL-1 receptor antagonist and inhibition of TNF-*α* and NF-*κ*B on endothelial cells [30]. The levels of adiponectin have been found to be inversely associated with marker of inflammation CRP in patients with diabetes [9] and coronary atherosclerosis [31]. However, no evidence suggests an association between plasma adiponectin and TNF-*α* in humans [32].

The relationship between adiponectin and OxS is likewise very complex. In general, low levels of adiponectin are associated with increased OxS [8], and some studies have concluded that adiponectin exerts its antiatherogenic and antidiabetic effects through the modulation of OxS [6]. In addition, higher levels of adiponectin are associated with more beneficial OxS profile in elderly population [12]. At the same time, positive as well as inverse correlations have been found between markers of OxS (isoprostanes) and adiponectin [6, 7]. An explanation for elevation of adiponectin in our allergic contact dermatitis patients may be that adiponectin represents a beneficial counter regulatory response to reduce oxidative burden similarly as it has been concluded in type 1 diabetes mellitus [8]. There was an inverse correlation between adiponectin and TPX, however, with the *P* value below the statistical significance (*P* = 0.06). Regardless of that, these results suggest the possibility that the increase of adiponectin level may be compensatory, evoked by high TPX concentration.

Leptin that is functionally and structurally related to proinflammatory cytokines [33] polarizes T cells towards a T_H_1 cell phenotype [11] while promoting monocyte recruitment and secretion of proinflammatory cytokines [33]. Thus, there are several mechanisms by which leptin could lead to greater inflammation. As women have markedly higher leptin concentrations as compared to men, even when adjusted for BMI [34], we investigated leptin levels only in female patients, comparing it to the value in female Co. After adjustment for BMI, we did not find differences in serum leptin levels between the patients and Co. There were no significant correlations between leptin levels and biomarkers of inflammation or OxS. Therefore, inclusion of adiponectin and leptin in severity assessment of allergic contact dermatitis may not be clinically useful.

In conclusion, the systemic effect of allergic contact dermatitis was most conspicuous in the parameters of OxS especially in TPX and OSI levels. The level of adiponectin was increased and showed a tendency to correlate inversely with TPX concentration. Therefore, as compared to inflammatory markers and adipokine levels, OxS parameters might be most helpful to assess disease activity and therapeutic response in allergic contact dermatitis.

## Figures and Tables

**Table 1:** The levels of inflammatory markers and oxidative stress characteristics in the patients with acute/subacute allergic contact dermatitis covering approximately 5% of body surface as compared to healthy controls.

	Allergic contact dermatitis (*n* = 40)	Healthy controls (*n* = 40)	*P*
BMI kg/m^2^	25.3 ± 5.5	24.8 ± 2.7∗/23.8 ± 3.4∗∗	—
hsCRP mg/L	1.96 ± 2.60	<1.0	ND
TNF-*α* pg/mL	6.32 ± 7.02	3.33 ± 0.95	*P* < 0.01
IL-6 pg/mL	1.34 ± 1.83	0.81 ± 0.30	*P* = 0.14
MCP-1 pg/mL	245.0 ± 105.1	190.9 ± 53.4	*P* = 0.059
IL-10 pg/mL	0.53 ± 0.30	0.68 ± 0.28	*P* < 0.05
TPX *µ*mol/L	719.9 ± 322.5	169.1 ± 58.8	*P* < 0.0001
TAC mmolTE/L	1.32 ± 0.19	1.55 ± 0.23	*P* < 0.0001
OSI %	55.9 ± 26.5	11.00 ± 3.7	*P* < 0.0001

BMI: body mass index; hsCRP: high sensitive C-reactive protein; TNF: tumor necrosis factor; IL: interleukin; MCP: monocyte chemoattractant protein; TPX: total peroxide concentration; TAC: total antioxidant capacity; TE: Trolox equivalent; OSI: oxidative stress index.

∗the value for cytokine control group.

∗∗the value for OxS control group.

ND: not done.

**Table 2:** Correlations between inflammatory and oxidative stress markers and their correlations with subjects' age and BMI in patients with allergic contact dermatitis and healthy controls.

	Patients and controls together (*n* = 120)	Patients with allergic contact dermatitis (*n* = 40)	Controls (*n* = 80)
BMI and age	*r* = 0.340***	*r* = 0.303	*r* = 0.541***
BMI and IL-6	*r* = 0.327**	*r* = 0.337*	*r* = 0.206
BMI and MCP-1	*r* = 0.295*	*r* = 0.380*	*r* = −0.167
BMI and TPX	*r* = 0.046	*r* = −0.011	*r* = 0.093
BMI and TAC	*r* = −0.084	*r* = −0.068	*r* = −0.093
IL-6 and MCP-1	*r* = 0.391***	*r* = 0.400*	*r* = 0.013
hsCRP and OSI	ND	*r* = 0.537***	ND
TPX and TAC	*r* = −0.380***	*r* = −0.040	*r* = −0.087

Correlations between baseline parameters were tested by Pearson rank correlation coefficient for data with a normal distribution. The *r* and *P* values are shown. Significant correlations are labeled as follows: *P < 0.05, **P < 0.01, and ***P < 0.001.

BMI: body mass index, TNF: tumor necrosis factor; IL: interleukin; MCP: monocyte chemoattractant protein; hsCPP: high sensitive C-reactive protein, TPX: total peroxide concentration; TAC: total antioxidant capacity; TE: Trolox equivalent; OSI: oxidative stress index; ND: not done.
